# The attack on November 13, 2015: organisation of the medico-judicial unit of the Hôtel-Dieu Hospital in Paris

**DOI:** 10.1080/20961790.2020.1768629

**Published:** 2020-05-28

**Authors:** Nicolas Soussy, Laurène Dufayet, Caroline Rey-Salmon, Charlotte Gorgiard

**Affiliations:** a Medico-Judicial Unit, Hôpital Hôtel-Dieu de Paris, APHP, Paris, France; b Centre AntiPoison et de Toxicovigilance, Hôpital Fernand Widal, APHP, Paris, France

**Keywords:** Forensic sciences, terrorism attacks, medico-judicial unit, November 13, 2015, total incapacity for work

## Abstract

The main aims of a medico-judicial unit are to ensure the examination of assault victims or persons in custody and to perform sampling necessary for investigations. Forensic examination is essential to describe the wounds and to evaluate the consequences of an assault by determining days of total incapacity for work (ITT). After the Paris attack on November 13, 2015, 121 victims were examined at the medico-judicial units of Paris. An initial forensic certificate was issued by forensic physicians with an assessment of physical ITT. A consultation with a forensic psychiatrist was systematically scheduled on the same day to obtain a forensic certificate for the psychological ITT. The average age of the victims was (33 ± 7) years and the sex ratio was 1.26. Most victims were in the Bataclan concert hall (78/121 or 64.5%). Of the 121 victims, 73 (60.3%) had projectile lesions (bullets, bolts and nuts, metal fragments, etc.) and 48 (39.7%) had non-projectile lesions (bruises, hematomas, etc.). The average physical ITT was 27 days (0; 190). The evaluation of the number of days of physical ITT was often complicated as some patients were still in medical care at the time of the initial examination. This experience enabled the Paris medico-judicial unit to anticipate the management required should any future event of this magnitude occur. It also reinforced cooperation between the medico-judicial unit and other departments, mostly emergency services and the forensic psychiatric unit. The Paris medico-judicial unit was thus able to offer a unique place of care by providing both physical and psychological examinations.

## Introduction

On November 13, 2015, a coordinated terrorist attack occurred in Paris and Saint-Denis, France. The first explosion took place outside the Stade de France in Saint-Denis while the French football team was playing in front of thousands of people. Then, a few minutes later, seven Parisian locations (six bars and the Bataclan concert hall) were targeted by terrorists using both explosive devices and firearms. A total of 130 people died, more than 300 were injured and nearly 2 000 were psychologically impacted [1, 2]. The massive influx of injured people presented an unprecedented situation in Paris and many hospitals in the area were involved [3–5]. Deceased person identifications and autopsies were carried out at the Paris Forensic Institute [6]. Forensic physicians were later in charge of medical forensic examinations of the surviving victims in the Paris medico-judicial unit in the Hôtel-Dieu Hospital. There are only a few studies in the medical literature on forensic management in the context of terrorist attacks, and all concern postmortem forensic medicine [7]. In this article, we offer a brief description of the organisation of the medico-judicial unit of the Hôtel-Dieu Hospital, Paris, before addressing the purposes and process of the medical forensic examinations carried out for surviving victims of the Paris terrorist attacks of November 13, 2015. The French literature on terrorist attacks mainly describes management of urgent medical responses [8, 9] and thanatological care [10]; no publications have described clinical forensic examinations of surviving victims. We also conduct a retrospective review of our files to present the main characteristics of these victims. Finally, we identify opportunities for improvement in managing such events.

## Organisation of the medico-judicial unit of the Hôtel-Dieu Hospital, Paris

The medico-judicial unit of the Hôtel-Dieu Hospital is one of 47 medico-judicial units in France, which are funded by the Ministry of Justice and were created in 1985 to meet the needs of magistrates and investigators in criminal matters [11].

The main aims of the medico-judicial units are to ensure the examination of victims of assault or persons in police custody, and to carry out the sampling necessary for investigations (toxicological or genetic). They also provide psychological support and information for victims and promote the development of forensic medicine through research and teaching. The medico-judicial unit of the Hôtel-Dieu Hospital in Paris is the largest medico-judicial unit in France owing to its volume of activity (about 30 000 acts per year). It is also the only medico-judicial unit in France to have a hospitalisation unit for patients in custody. Another particularity of this unit relates to victim examination. The main purpose of a forensic examination is to describe the wounds and evaluate the physical and psychological consequences of an assault by determining days of total incapacity for work (ITT). ITT is a legal concept that enables a judge handling a case to assess the severity of injuries inflicted on the victim. In French criminal law, an ITT duration of over 8 days is one of several criteria (another criterion is aggravating circumstances) for the delictual characterisation of the offense [3]. It is determined by the duration of the real and overall discomfort experienced by the victim when performing everyday activities. In the Hôtel-Dieu medico-judicial unit, physical examinations are conducted by a forensic physician and psychological examinations are conducted in a separate consultation by a psychiatrist trained in forensic practice. This permits a more detailed evaluation of the psychological consequences of the assault. Two certificates are made out with two separate numbers of ITT days.

The medico-judicial unit team consists of forensic physicians, physicians of other specialties with training in forensics, nurses, caregivers and psychologists. The Paris medico-judicial unit also benefits from the presence of volunteers from Paris Aide aux Victimes, a French association that supports victims of crime.

In addition to these units, the Medico-Psychological Units (CUMP), which were created after the 1995 Paris terrorist attacks, provide initial psychological care for victims of traumatic events. The creation of CUMP is a milestone in victimology in France and its interface with medico-judicial units is essential as it creates a care network for victims.

## Purposes and process of the medical forensic examination of victims of the attacks on November 13, 2015

The day after the attacks, the anti-terrorist section of the Paris public prosecutor’s office requested the medico-judicial unit of the Hôtel-Dieu Hospital to examine surviving victims.

As for any assault victim, the main purpose of this medical forensic examination was to describe the wounds and to evaluate the physical consequences of the assault by determining ITT days. In the case of a terrorist attack, the offense is criminal, regardless of the number of ITT days. In addition to its judicial role, ITT may help victims to assert their rights, with the acknowledgment of their status as victims of terrorism, and to obtain compensation from a specific guarantee fund. In this context, the objectives of the consultation were also to identify the injury mechanisms and the ballistic trajectories.

Appointments were made over the phone by police officers. Upon arrival at the medico-judicial unit, the victim was welcomed into the examination room by both a doctor and a nurse. At the beginning of the consultation, the victim recollected the events. The victim was then invited to list all their psychological problems related to the attack. Initial medical documents related to the care of the victim were then transcribed onto the certificate. A physical examination was performed to assess any physical pain or discomfort. In some cases, photographs were taken for inclusion in the certificate, with the victim’s agreement, to illustrate ballistic trajectories.

A forensic certificate was produced by the service’s forensic physicians with an assessment of the physical ITT. Physicians were invited to use a standard unit certificate framework for the redaction of their certificates. Then, on the same day, victims were examined by a psychiatrist, who established the psychological impact of the event and produced a separate forensic certificate [12]. These two certificates were sent by mail to the anti-terrorist section of the Paris public prosecutor’s office. Forensic physicians saved their certificates to a specific file on the medico-judicial unit computer network.

Another aim of the consultation, in addition to the issuing of certificates, was to refer victims to care facilities or victim support associations. Outside the framework of the judicial procedure, the medico-judicial unit has a unique role in victim information and orientation. In Paris, the presence of volunteers from associations greatly facilitates victims’ access to information. They offer support with administrative procedures, but also psychological follow-up if needed. After the Paris attack, such volunteers helped many victims to compile their files and obtain compensation *via* a specific guarantee fund.

The authorities’ decision to entrust the medico-judicial unit of Paris with the victims made it possible to centralise and homogenise the production of forensic certificates. The Paris medico-judicial unit has thus been able to offer a unique place of care in addition to psychological and physical examinations [13].

## Characteristics of victims of the attacks of November 13, 2015, examined at the medico-judicial unit of the Hôtel-Dieu Hospital

We performed a retrospective search of all the files saved to the relevant file of the medico-judicial unit computer network.

Between November 14, 2015 and July 28, 2016, 121 victims of the Paris attack on November 13, 2015 were examined at the Paris medico-judicial unit with the average age of (33 ± 7) years (mean ± SD) (grouped by age: 15–29, *n* = 47; 30–44, *n* = 62; 45–59, *n* = 12). The victims were mainly in the Bataclan concert hall at the time of the attacks (78/121 or 64.5%) (Table 1). The sex ratio was 1.26 (male:female) and the average time between attacks and consultations was 51.7 days, ranging from 4 to 258 days. Physical lesions were classified as “projectile” lesions (bullets, bolts and nuts, metal fragments, blasts) and “non-projectile” lesions (bruises, hematomas, superficial wounds, fractures related to crowd movements). Of the 121 victims examined, 73 (60.3%) had projectile lesions. Many victims had more than one projectile lesion, and a total of 100 projectile lesions were identified. The remaining victims examined presented non-projectile lesions (48 or 39.7%). The lesions were mainly bruises or hematomas, superficial wounds or fractures. Overall, the average physical ITT was 27 days, ranging from 0 to 190 days. Of the 121 people examined, a physical ITT greater than 8 days was determined in 60 cases (49.6%).

**Table 1.** Place of the attack.

Place of the attack	Total (*N* = 121)
Bataclan	78
Le Carillon	6
Stade de France	4
Le Petit Cambodge	5
La Belle Equipe	4
Comptoir Voltaire	3
La Bonne Bière	1
Casa Nostra	1
Unknown	19

## Discussion and areas for improvement

First, all medical examiners were forensic physicians and were assisted by a nurse; both physician and nurse were experienced in victimology. This permitted the issuing of quality certificates with a standard framework. The medico-judicial unit of the Hôtel-Dieu Hospital was able to adapt to a sudden increase in the number of daily patients in the days following the attack. This would not have been possible without the participation of every member of the team. The consultation with the psychiatrist was systematically scheduled on the same day as the physical examination, and the dispatch of forensic certificates to the requesting service was both quick (*via* mail) and centralised.

Nevertheless, difficulties were encountered with this system. From a forensic perspective, physicians were confronted with complex injury mechanisms. Many patients were wounded by unusual projectiles (e.g. bolts and nuts) and had undergone major surgical interventions. In comparison, the most common injury patterns of the Boston Marathon bombing victims in April 2013 were secondary blast injuries [14, 15] owing to the nature of the improvised explosive device: pressure cookers filled with explosive devices, ball bearings, nails and screws. These injuries were mostly located on the lower limbs; only 10.4% were on the head and neck region, probably because the bomb was on the ground and the explosion was of low intensity.

Fortunately, most patients had initial medical certificates, which combined with their recollection of the event, helped the forensic physician to comprehend and evaluate the injury mechanism involved. The evaluation of the number of days of physical ITT was often complicated by the fact that some patients were still in medical care at the time of the initial examination. These patients were informed of the opportunity to return for a secondary consultation at the end of their medical care so that a complementary certificate with a final number of physical ITT days could be issued. Some victims benefited from a forensic evaluation in other medico-judicial units close to their place of residence. The lack of coordination between the different French medico-judicial units made it impossible to compare care or to reflect at the national level on the role of the medico-judicial units for surviving victims of terrorist events. Finally, a lack of medico-judicial training for terrorist attacks meant that the Paris medico-judicial unit was not involved in the initial care of the victims, particularly in the collection and preservation of ballistic elements collected by the emergency teams. Forensic pathologists have specific knowledge that could be useful to clinicians, justice teams and victims in initial stages of these kinds of dramatic events [16].

## Conclusion

In terms of both the number of victims and the diversity of lesions, this terrorist attack presented a major challenge to the medico-judicial unit of the Hôtel-Dieu Hospital in Paris. However, this experience will help to anticipate the management required in any future events of this magnitude and to develop and improve cooperation with emergency and psychiatric services. Future studies could additionally investigate psychological management as well as victim examinations carried out in other centres.
